# Supplementary material for: β-sitosterol alleviated HFD-induced atherosclerosis by regulating the MAPK/Nrf2/NLRP3 pathway in ApoE−/− mice
Source: PLoS One. 2025 Sep 18;20(9):e0332990. doi: 10.1371/journal.pone.0332990 (PMC12445543; doi:10.1371/journal.pone.0332990)
Supplement: Attachment — Submitted filename: Response to Reviewers.docx [file pone.0332990.s002.docx]

Response to Reviewer Comments

Manuscript ID: PONE-D-25-25290

Title:β-sitosterol alleviated HFD-induced atherosclerosis by regulating the MAPK/Nrf2/NLRP3 pathway in ApoE−/− mice

Dear Pro. Yang,

We sincerely appreciate the reviewers for their time and constructive comments, which have helped us improve the quality of our manuscript. Below, we provide a point-by-point response to each comment. All changes in the revised manuscript are highlighted in yellow changes for easy reference.

Journal Requirements:

Comment 1. Please ensure that your manuscript meets PLOS ONE's style requirements, including those for file naming.

Reply: The manuscript meets PLOS ONE's style requirements, including those for file naming.

Comment 2. To comply with PLOS ONE submissions requirements, in your Methods section, please provide additional information regarding the experiments involving animals and ensure you have included details on (1) methods of sacrifice, (2) methods of anesthesia and/or analgesia, and (3) efforts to alleviate suffering.

Reply: Thank you for pointing out this problem in manuscript. The additional information has been provided in Methods section in line 99-100 and line 108-109 with changes highlighted in yellow.

Comment 3. Please also include the statement “There was no additional external funding received for this study.” in your updated Funding Statement. Please include your amended Funding Statement within your cover letter.

Reply: We appreciate for your valuable comment. The Funding Statement has been updated in cover letter with changes highlighted in yellow.

Comment 4. Please confirm at this time whether or not your submission contains all raw data required to replicate the results of your study.

Reply: All relevant data are within the manuscript.

Reviewer #1

Comment 1. Sample size justification:

While using 6 mice per group is common, it would strengthen the study to briefly justify this choice. Was any power analysis performed?

Reply: We appreciate the reviewer’s suggestion. The sample size of 6 mice per group was determined based on common practice in similar studies and ethical considerations to minimize animal use while ensuring statistical reliability. We have added a justification in the Methods section with changes highlighted in yellow (Lines 98-100) to clarify this point.

Comment 2. Statistical reporting:

The use of ANOVA is appropriate, but I recommend reporting exact p-values where possible, instead of ranges (e.g., “p < 0.05”), to improve transparency and reproducibility.

Reply: We thank the reviewer for this constructive suggestion. In the revised manuscript, we have now reported exact *p*-values for all ANOVA results in Figure 1-3 where applicable.

Comment 3. NF-κB interpretation:

You mention that β-sitosterol had no significant effect on NF-κB levels. Given the central role of NF-κB in inflammation, a short paragraph discussing this finding—and how it fits with the MAPK/Nrf2/NLRP3 interplay—would be appreciated.

Reply: We appreciate the reviewer’s insightful observation regarding the role of NF-κB in inflammation. A short paragraph has been added in line 260-264 with changes highlighted in yellow to discuss this finding.

Comment 4. Mechanistic depth:

The proposed mechanism is biologically plausible and compelling. However, it would be good to acknowledge the limitations of relying on expression data alone. For future work, pathway inhibition or knockout studies would help confirm causal links between β-sitosterol and the signaling cascades.

Reply: We sincerely appreciate the reviewer's insightful suggestion regarding mechanistic validation. We fully agree that while our expression data support the proposed MAPK/Nrf2/NLRP3 interplay, functional studies are crucial for establishing causality (in line 265-266 with changes highlighted in yellow).

Comment 5. English and grammar.

Reply: Thank you so much for your careful check. Some sentences have been modified in line 56 and line 86-87 with changes highlighted in yellow.

Comment 6. Ethical approval:

You clearly mention ethical approval by the China Pharmaceutical University Animal Care Committee. If available, please include the approval number.

Reply: The approval number has been added in line 98 with changes highlighted in yellow.

Reviewer #2

Comment 1. The expression patterns of MAPK/Nrf2/NLRP3 signaling pathway and MAPK/Nrf2/NLRP3 pathway remain consistent.

Reply: We appreciate the reviewer’s insightful comment. To improve clarity, we have unified the terminology to “MAPK/Nrf2/NLRP3 pathway” in line 45 with changes highlighted in yellow throughout the revised manuscript.

Comment 2. p38 MAPK: On line 167, it is written as p38 MAPK, while elsewhere (such as line 124, Figure 2B) it is p38 MAPK. Should be unified as p38 MAPK (with spaces)

Reply: Thank you so much for your careful check. p38MAPK has been modified with spaces in line 169 with changes highlighted in yellow.

Comment 3. Figures 1F-G: The caption (line 160) states (n=3). However, the results section (line 155) describes the aortic root plaques, and the methods section (lines 109-113) also describes section staining of the aortic root. Each group has 6 animals, and usually aortic root section analysis is performed on multiple sections, but the final statistical unit is usually the number of animals per group. The caption (n=3) contradicts the animal grouping of n=6.

Reply: We sincerely appreciate the reviewer’s attention to this important detail. In this study, although 6 animals per group were initially included, technical challenges during sample processing resulted in only 3 high-quality datasets per group meeting our pre-defined inclusion criteria. Future studies will prioritize optimized tissue fixation protocols to minimize attrition.

Comment 4. Figure 3I: The result description (lines 193-194) mentions scale bar=100 μ m, but the scale is not shown on the figure. A ruler must be added to the diagram.

Reply: We appreciate the reviewer’s careful attention to detail. We apologize for the oversight and have now added a clearly visible 100 μm scale bar to Figure 3I in the revised manuscript. This ensures the figure meets publication standards for accurate representation of scale.

Comment 5. Figures 1, 2, and 3: The statistical significance markers (*, *, * * *, * * *, ns) in all the captions cannot be seen on the corresponding data points or whether there are markers on the bar chart. It is necessary to confirm whether these significant markers have been correctly labeled at the corresponding comparison positions in the graph. Reviewers need to be able to directly see which comparisons are significant from the graph.

Reply: We sincerely appreciate the reviewer’s meticulous critique regarding the presentation of statistical significance in our figures. In the revised manuscript, we have added explicit P-values (in parentheses) to all figure captions to complement the asterisk notation.

Comment 6. Line 229, the sentence, “For example, Camosic acid downregulated Nrf2, resulting in a decrease expression of antioxidant enzymes such as CAT”, should be added in here and its reference, https://doi.org/10.26599/FMH.2025.9420021added in here.

Reply: 3. We sincerely appreciate the reviewer's constructive suggestions. The sentence and its reference have been added in line 233-234 with changes highlighted in yellow.
