# Supplementary material for: β-sitosterol alleviated HFD-induced atherosclerosis by regulating the MAPK/Nrf2/NLRP3 pathway in ApoE−/− mice
Source: PLoS One. 2025 Sep 18;20(9):e0332990. doi: 10.1371/journal.pone.0332990 (PMC12445543; doi:10.1371/journal.pone.0332990)
Supplement: Attachment — Submitted filename: Response_to_Reviewers_auresp_2.docx [file pone.0332990.s003.docx]

Response to Reviewer Comments

Manuscript ID: PONE-D-25-25290

Title:β-sitosterol alleviated HFD-induced atherosclerosis by regulating the MAPK/Nrf2/NLRP3 pathway in ApoE−/− mice

Dear Pro. Yang,

We sincerely appreciate the reviewers for their time and constructive comments, which have helped us improve the quality of our manuscript. Below, we provide a point-by-point response to each comment. All changes in the revised manuscript are highlighted in yellow changes for easy reference.

Journal Requirements:

Comment 1. If the reviewer comments include a recommendation to cite specific previously published works, please review and evaluate these publications to determine whether they are relevant and should be cited. There is no requirement to cite these works unless the editor has indicated otherwise.

Reply: Thank you for pointing out this problem in manuscript. The sentence and its reference have been deleted.

Comment 2. Please review your reference list to ensure that it is complete and correct.

Reply: We thank the editor for bringing this to our attention. We confirm that no retracted articles were cited in our manuscript. All references are current, relevant, and correctly presented.

Reviewer #1

Comment 1. Ensure all histological figures include scale bars for clarity.

Reply: We appreciate the reviewer’s suggestion. We have added a clearly visible 500 μm scale bar to Figure 1F in the revised manuscript.

Comment 2. In the Discussion, explicitly mention the limitation regarding the absence of release/penetration profile studies in the topical formulation.

Reply: We thank the reviewer for this constructive suggestion. The Strengths does not align with the submitted version of our manuscript. For instance, "Hibiscus sabdariffa" and "Table 7" have not existed in our manuscript. Besides, this suggestion: “In the Discussion, explicitly mention the limitation regarding the absence of release/penetration profile studies in the topical formulation” dose not align with our manuscript. Given this inconsistency, we wondered if the reviewer might have inadvertently referred to different draft of the manuscript when formulating their comments. Thank you for your time and guidance.
